# Mitochondrial Dysfunction in Skeletal Muscle of a Fibromyalgia Model: The Potential Benefits of Melatonin

**DOI:** 10.3390/ijms20030765

**Published:** 2019-02-11

**Authors:** Gaia Favero, Francesca Bonomini, Caterina Franco, Rita Rezzani

**Affiliations:** 1 Anatomy and Physiopathology Division, Department of Clinical and Experimental Sciences, University of Brescia, Viale Europa 11, 25123 Brescia, Italy; gaia.favero@unibs.it (G.F.); francesca.bonomini@unibs.it (F.B.); caterinafranco.1996@gmail.com (C.F.); 2 Interdipartimental University Center of Research “Adaption and Regeneration of Tissues and Organs-(ARTO)”, University of Brescia, 25123 Brescia, Italy

**Keywords:** fibromyalgia, skeletal muscle, mitochondria, oxidative stress, melatonin

## Abstract

Fibromyalgia syndrome (FMS) is considered a musculoskeletal disorder associated to other symptoms including chronic pain. Since the hypothesis of FMS etiogenesis is consistent with mitochondrial dysfunction and oxidative stress, we evaluated the pathophysiological correlation among these factors studying some proteins involved in the mitochondrial homeostasis. We focused our attention on the roles of peroxisome proliferator activated receptor gamma coactivator-1alpha (PGC-1α), mitofusin2 (Mfn2), and coenzyme Q10 (CoQ10) in reserpine-induced myalgic (RIM) rats that manifest fibromyalgia-like chronic pain symptoms. First, we underlined that RIM rats are a good model for studying the pathophysiology of FMS and moreover, we found that PGC-1α, Mfn2, and CoQ10 are involved in FMS. In fact, their expressions were reduced in gastrocnemius muscle determining an incorrect mitochondrial homeostasis. Today, none of the currently available drugs are fully effective against the symptoms of this disease and they, often, induce several adverse events; hence, many scientists have taken on the challenge of searching for non-pharmacological treatments. Another goal of this study was therefore the evaluation of the potential benefits of melatonin, an endogenous indoleamine having several functions including its potent capacity to induce antioxidant enzymes and to determine the protective or reparative mechanisms in the cells. We observed that melatonin supplementation significantly preserved all the studied parameters, counteracting oxidative stress in RIM rats and confirming that this indoleamine should be taken in consideration for improving health and/or counteract mitochondrial related diseases.

## 1. Introduction

Fibromyalgia syndrome (FMS) is a chronic musculoskeletal pain disorder associated to other symptoms that are very difficult to identify [1]. This pathological condition affects about 3–10% of the population; its incidence is prevalent in women than in men [2], and it represents a diagnostic challenge for clinicians [3].

The hypothesis of FMS etiogenesis is consistent with mitochondrial dysfunction and oxidative stress even if the pathophysiological relationship among them remains unclear [4]. Many authors suggested that loss of mitofusin2 (Mfn2), an outer mitochondrial membrane protein which mediates the fusion of mitochondria [5], causes depletion of mitochondrial coenzyme Q10 (CoQ10), which in turn leads to respiratory chain dysfunction by altering mitochondrial uncoupling proteins, mitochondrial permeability transition pore and reactive oxygen species (ROS) production [6]. Moreover, CoQ10 regulates serotonin levels and depressive symptoms in fibromyalgic patients [7]. The role of Mfn2 in regulating CoQ10 for optimal function of the mitochondrial respiratory chain is not well investigated; however, the study of Mourier et al. [6] unravels an unexpected and novel role of Mfn2 in maintenance of the terpenoid biosynthesis pathway, which is necessary for mitochondrial coenzyme Q biosynthesis. Moreover, other studies found that the peroxisome proliferator-activated receptor gamma coactivator-1alpha (PGC-1α) signaling pathway is able to modulate Mfn2 gene and protein expression [8], thus determining a shift in the focus of the majority of the studies from etiology to symptom management.

To our knowledge, reserpine-induced myalgic (RIM) rats are the only animal model mimicking FMS characteristics as reported by Nagakura et al. [9]. Thus, the objective of this study was to stress the contribution of RIM rat model for understanding the pathophysiology of FMS, considering firstly myogenin which is an important protein for metabolic pathway of skeletal muscle, and then evaluating the roles of PGC-1α, Mfn2, CoQ10, and mitochondrial involvement in this disorder.

Today, none of the currently available drugs are fully effective against the whole spectrum of FMS symptoms and they often induce several adverse events [10,11,12]. Many clinical trials showed that most patients with FMS show defects in melatonin secretion and this might explain the complaint of sleep disturbance [13]. Many authors reported the therapeutic usefulness of melatonin in different fields of medicine, e.g., the treatment of FMS suggesting that it is an alternative and safe treatment for patients with this pathology since it determines an improvement in severity of pain [14,15,16]. Moreover, serum levels of melatonin and its precursors were reported to be low in patients with FMS affecting sleep and perception and therefore, the melatonin supplementation, responsible for reducing the oxidative stress burden during aging and/or pathological conditions [16,17,18], may be a novel approach for the management of patients with FMS [19,20]. Furthermore, considering the oxidative damage associated with FMS, the use of powerful antioxidants such as melatonin, alone or in combination with other therapies, may improve the outcome of this pathology [21,22,23]. 

To date, the mechanism by which melatonin can be effective against FMS is not well-known, further research is required to clarify whether melatonin is useful for FMS treatment/prevention. In our previous paper we showed, in myalgic rats, that melatonin increased antioxidant enzymes, such as superoxide dismutase 1 and catalase, which were reduced in this disease [23]. At this regard, the other aim of this study was to investigate the potential melatonin mechanism(s) of action mediated by the oxidative stress, as mentioned above. Moreover, it is important to remember that we had previously observed that melatonin supplementation in RIM animal model induced anti-inflammatory, antioxidant, and analgesic responses at skeletal muscle level [23], but we had not considered or evaluated the correlation between the mitochondrial dysfunction and many of the involved factors such as PGC-1α, Mfn2, and CoQ10.

## 2. Results

As reported in our previous study [23], the experimental animals only treated with melatonin or reserpine/melatonin vehicles showed no significant differences compared with untreated control rats, and therefore these experimental groups are defined generically as “control (CTR)” in the following analysis description.

### 2.1. Spontaneous Locomotor Activity Monitoring

**Figure 1:**
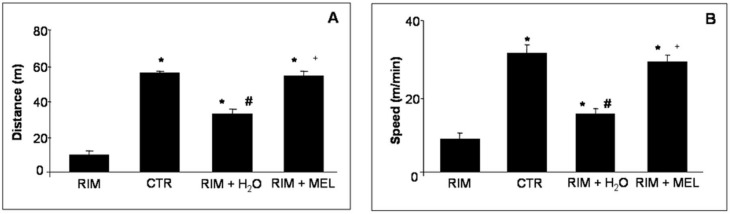
Spontaneous locomotor activity. The graph summarizes the distance travelled (**A**) and the speed (**B**) of spontaneous locomotor activity. * *p* ≤ 0.05 vs. RIM + H_2_O; # *p* ≤ 0.05 vs. CTR; + *p* ≤ 0.05 vs. RIM + H_2_O. CTR: control; H_2_O: tap water; MEL: melatonin; RIM: reserpine-induced myalgia.

The RIM animals are a valid model of FMS, also because their voluntary motor activity is significantly reduced compared with controls. In brief, the RIM group showed both a lower running distance traveled and a reduced running speed compared to control rats. The treatment with melatonin of RIM rats improved significantly their voluntary motor activity, increasing both distance travelled and speed of locomotor activity, and reaching values comparable to control rats. It is important to underline that also RIM rats plus tap water for two months showed a progressive improvement in voluntary running activity, anyway the distance traveled and the activity speed were lower compared with the RIM group treated with melatonin (Figure 1).

### 2.2. Morphological Evaluations of Gastrocnemius Muscle

**Figure 2:**
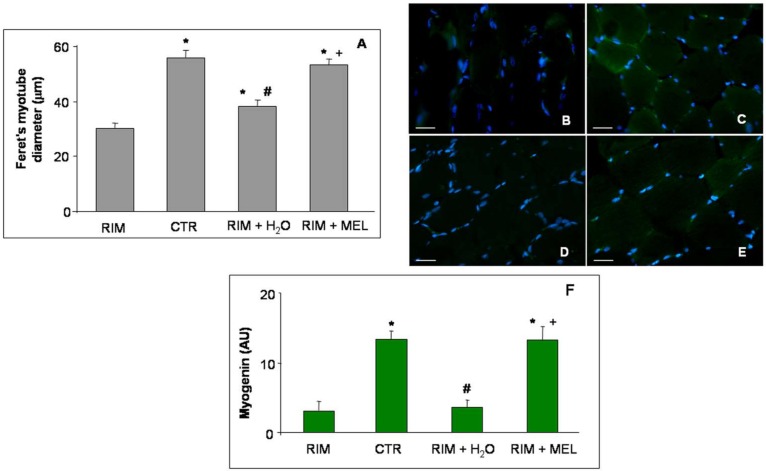
Myotube diameter and myogenin expression. The graph (**A**) shows the analyses of Feret’s myotube diameter of gastrocnemius skeletal muscle, expressed in µm. The immunofluorescence photomicrographs (**B**–**E**) show the gastrocnemius skeletal muscle myogenin expression of RIM rats (**B**), control rats (**C**), RIM rats plus tap water (**D**), and RIM rats supplemented with melatonin (**E**). Bar equal: 20 µm. Graph (**F**) summarizes the immunomorphometrical measurement of myogenin immunopositivity. * *p* ≤ 0.05 vs. RIM; # *p* ≤ 0.05 vs. CTR; + *p* ≤ 0.05 vs. RIM + H_2_O. CTR: control; H2O: tap water; MEL: melatonin; RIM: reserpine-induced myalgia.

As expected from the RIM experimental group, these animals showed a significant skeletal muscle atrophy, as reported previously [23], and as also confirmed in this study by evaluating the myotube diameter. In detail, the Feret’s diameter of gastrocnemius myotubes decreased in the RIM group with respect to control rats. Interestingly, RIM rats plus tap water showed a weak increase in myotube diameter, which was however lower compared to the myotube diameter of RIM rats treated with melatonin; this shows that melatonin supplementation prevents the reduction of myotube diameter (Figure 2A).

Furthermore, we also investigate the expression of a myogenic transcription factor and regulator of muscle regeneration: myogenin [24]. Myogenin (green staining) was very weakly expressed in the gastrocnemius of the RIM group (Figure 2B) with respect to control rats, which showed a moderate/strong gastrocnemius expression of this myogenic transcription factor (Figure 2C). The RIM group plus tap water showed a very weak myogenin expression (Figure 2D), although the RIM group treated with melatonin showed a significant increase of myogenin expression, reaching control group level (moderate/strong expression) (Figure 2E). These observations are also confirmed by the immunomorphometry analyses plotted in Figure 2F.

### 2.3. Mitochondrial Markers Evaluation

**Figure 3:**
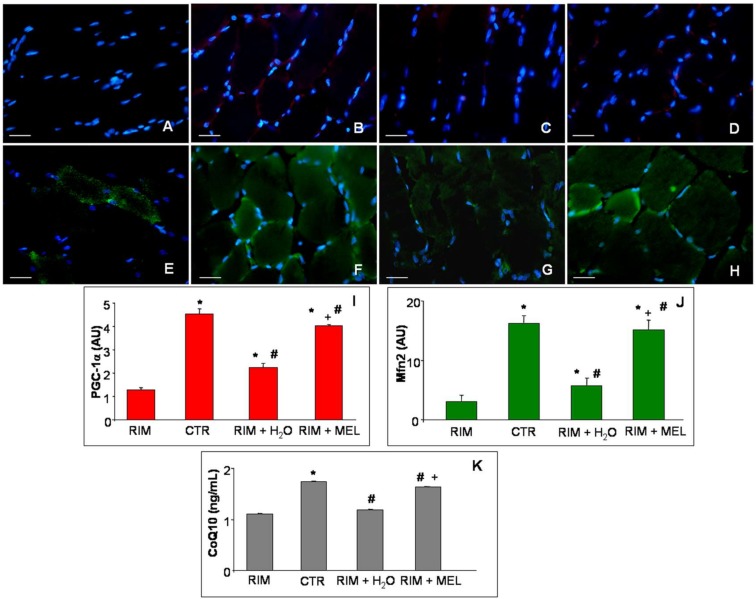
Gastrocnemius mitochondrial markers evaluation. Immunofluorescence photomicrographs of peroxisome proliferator activated receptor gamma coactivator-1alpha (**A**–**D**) and mitofusin2 (**E**–**H**) expression in gastrocnemius skeletal muscle of RIM rats (**A**,**E**), control rats (**B**,**F**), RIM rats plus tap water (**C**,**G**) and RIM rats supplemented with melatonin (**D**,**H**). Bar equal: 20 µm. The graphs summarize the immunomorphometrical measurement of peroxisome proliferator activated receptor gamma coactivator-1alpha (**I**) and mitofusin2 (**J**) immunopositivities. The graph (**K**) summarizes gastrocnemius coenzyme Q10 level of all experimental groups. * *p* ≤ 0.05 vs. RIM; # *p* ≤ 0.05 vs. CTR; + *p* ≤ 0.05 vs. RIM + H_2_O. CoQ10: coenzyme Q10; CTR: control; H_2_O: tap water; MEL: melatonin; Mfn2: mitofusin2; PGC-1α: peroxisome proliferator activated receptor gamma coactivator-1alpha; RIM: reserpine-induced myalgia.

Gastrocnemius expressions of PGC-1α (Figure 3A–D; red staining) and Mfn2 (Figure 3E–H; green staining) were evaluated to better investigate the mitochondrial alterations involved in fibromyalgic skeletal muscle dysfunctions, including through ultrastructural evaluation as reported in previous papers [23,25]. In detail, we observed that PGC-1α gastrocnemius expression was almost null in RIM group (Figure 3A), whereas in control rats it was moderately expressed and localized in the cytoplasm of interstitial cells, outside the gastrocnemius skeletal muscle fibers (Figure 3B). The RIM group plus tap water showed an absent/very weak expression of PGC-1α (Figure 3C), which was moderately expressed in the RIM group treated with melatonin (Figure 3D). Remarkably, since PGC-1α may modulate Mfn2 [8], the gastrocnemius of RIM group showed a weak Mfn2 expression (Figure 3E) against a moderate/strong expression in control rats (Figure 3F). The RIM group plus tap water showed a weak increase of Mfn2 (weak/moderate expression) (Figure 3G), which was higher in the RIM group treated with melatonin (moderate/strong expression) (Figure 3H). All these observations are also confirmed by the immunomorphometrical quantifications plotted for PGC-1α in Figure 3I and for Mfn2 in Figure 3J.

Finally, due to the strict correlation between Mfn2 depletion and CoQ10 reduction [6], we evaluated also the level of CoQ10 gastrocnemius muscle samples. Skeletal muscle CoQ10 level was reduced in the RIM group compared to controls and weakly increased in the RIM group plus tap water. Interestingly, RIM supplemented with melatonin group showed a significant increase of CoQ10 concentration, reaching values comparable to controls. Figure 3K summarizes the gastrocnemius CoQ10 concentration in each experimental group studied.

## 3. Discussion

Herein, we demonstrated that: (1) RIM rats are a good model for evaluating the etiogenesis of FMS; (2) mitochondrial dysfunction and the oxidative stress mediated by PGC-1α, Mfn2 and CoQ10 are involved in the FSM outcome; (3) melatonin is useful to improve mitochondrial performance.

Previously, both our and other studies demonstrated that RIM rats showed a reduction in locomotor activity and a decrease in body weight with significant aversion of eating [23,26]. These findings are consistent with the symptoms identified in the FMS [27,28], and were thus a starting point for the other objectives of this study.

Interestingly, the obtained results demonstrate that, after spontaneous exercise carried out every day, control rats showed a moderate/strong expression of myogenin. This finding is consistent with other data underlining the skeletal muscle adaptation, mediated by the increase of many protein expressions as a response to different activities. Moreover, several studies suggested that myogenin is, together with other myogenic factors, the key player in the process of prenatal and postnatal myogenesis [29]. Prenatally, myogenin and the other myogenic factors are expressed only in progenitor cells and myoblasts but, postnatally, they are important to regulate the myogenesis process and physical performance via satellite cells [30]. For this reason, they are widely recognized for their contribution in maintaining muscle mass, besides their having an important role in guaranteeing muscle regeneration and hypertrophy during life span [31]. The muscle, in fact, modifies its own metabolism in order to set it on the basis of different stimuli; on the contrary, we showed that RIM rats had very weak expression of myogenin because these animals had pain and loss of muscle mass that impaired the ability to perform movements and exercise. This could lead to a reduction of the adaptation ability of skeletal muscle.

In the framework of our search for a correlation between myogenin expression and exercise, these results taken together showed that prolonged periods of inactivity lead to alterations in skeletal muscle with increased production of ROS. This suggests that oxidative stress could be a major trigger for muscle disease [32].

Moreover, mitochondria play a critical role in the regulation of many signaling pathways controlling muscle mass and an imbalance of mitochondrial dynamics induces production of ROS and several other oxidative-associated factor(s) production such as PGC-1α and Mfn2 [33,34]. For this purpose, our results showed a moderate and moderate/strong expression respectively of PGC-1α and Mfn2 in control rats, whereas their expression was seriously reduced (weak/very weak) in RIM rats.

It is known that PGC-1α is an important factor controlling mitochondrial shape, content and biogenesis [35,36,37]. Moreover, its higher levels are critical and important for maintaining the mitochondrial functions as well as for improving muscle wasting, as recently reported by Hyatt et al. [38]. Our data support and suggest that PGC-1α is a master regulator of skeletal muscle functions linked to Mfn2.

As regards Mfn2, it is an outer mitochondrial membrane GTPase and it is important for mitochondrial fusion, which in turn affects mitochondrial dynamics and functions. Moreover, the alterations of Mfn2 expression modify cell respiration and oxidative phosphorylation subunit expression in both cultured non-muscle and muscle cells [39]. It is known that PGC-1α participates in the stimulation of Mfn2 expression under a variety of conditions characterized by enhanced energy expenditure [40]. Therefore, our findings expand these data underlining that, if the expression of PGC-1α is very low, mitochondria have dysfunctions and Mfn2 expression is very low too, and finally, skeletal muscle is not in good health and its functions are impaired.

Considering all data reported above, mitochondria are dynamic organelles whose functions are essential for maintaining of protein homeostasis in health and disease, in many tissues, including skeletal muscle [41]. For corroborating these results, we evaluated the expression of another marker of mitochondrial functions such as CoQ10, which is not expressed in patients with FMS, as many authors reported [42,43,44]. We demonstrated the reduction of CoQ10 expression in the skeletal muscle of RIM rats compared to control animals, and for this reason its supplementation could improve the clinical symptoms associated with this disease, as reported by Cordero et al. [1].

**Figure 4:**
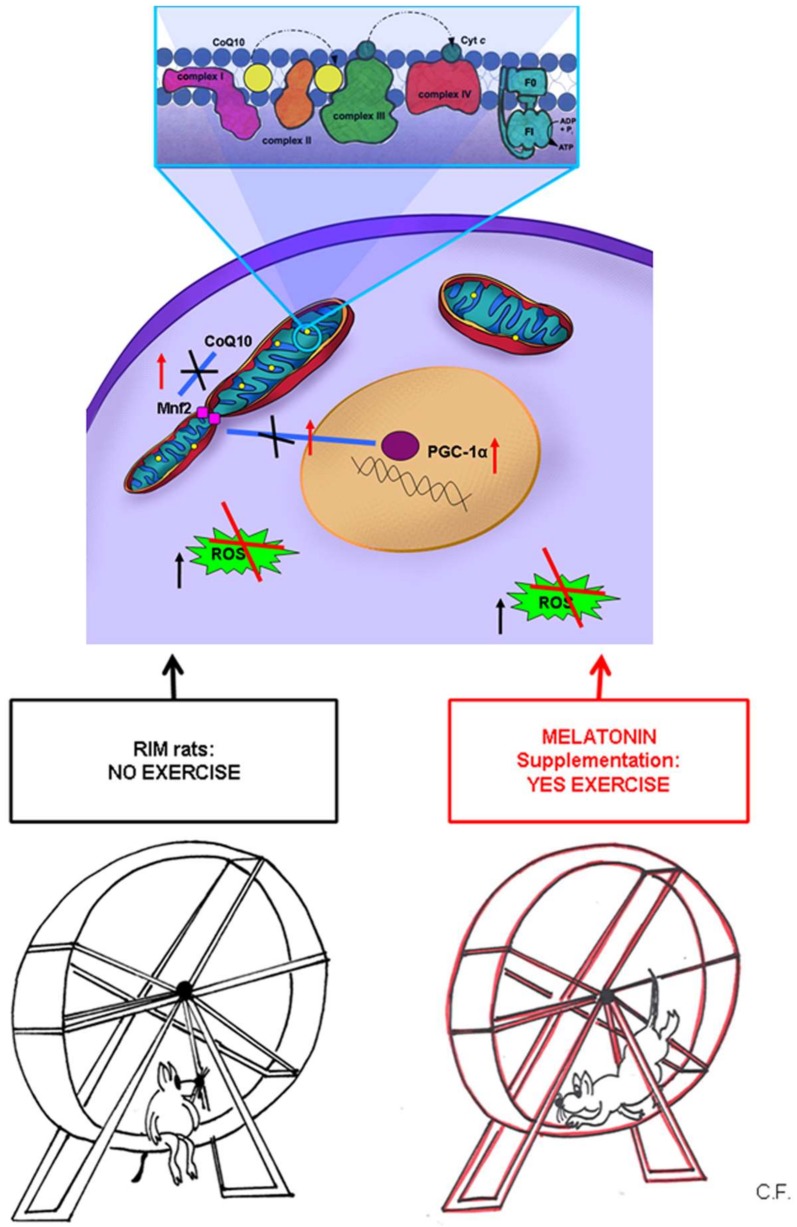
Mitochondrial involvement in fibromyalgia syndrome. A schematic diagram representing the important role of mitochondria in normal and in FMS underlining the biosynthetic pathway mediated by PGC-1α. The decrease of PGC-1α expression induces downregulation of Mfn2 that, in turn, determined a decrease of CoQ10 expression in the inner of mitochondrial membrane. In mitochondria CoQ10 is a part of electron transport chain among several complexes likely to cytochrome C on the complex IV. It is also depicted the potential beneficial effects of melatonin in RIM rats and so, its role in the maintenance of health. Mfn2 = mitofusin2; PGC-1α = peroxisome proliferator-activated receptor gamma coactivator 1-alpha; ROS = reactive oxygen species; CoQ10 = coenzyme Q10; Cyt c = cytochrome complex; RIM rats = reserpine-induced myalgic rats; F0 = “Fraction 0” of the ATPase; it is a proton pore that is embedded in the mitochondrial membrane; F1 = “Fraction 1” of the ATPase; it is the portion responsible for hydrolyzing ATP; ADP = adenosine diphosphate; Pi= inorganic phosphate; ATP = adenosine triphosphate.

Figure 4 summarizes the data reported above, indicating that we hypothesize the important role of mitochondria in FMS and the biosynthetic pathway mediated by PGC-1α.

The last but not least important objective of this study was to evaluate whether complementary and/or alternative medical treatments could improve the outcome of this disorder considering that pharmacological interventions give variable benefits and common side effects [12,45]. For this purpose, we stressed the potential beneficial effects of melatonin in RIM rats studying the mechanisms by which this indoleamine acts. Melatonin is the major biologically active molecule secreted by the pineal gland and has several functions, including its potent capacity to induce antioxidant enzymes and to determine protective or reparative mechanisms in the cells [46]. Since melatonin has the properties reported above, it is also important to remember that Suofu et al. [47] demonstrated that melatonin is synthesized in the mitochondria that are the major site of free radical generation. This point is very important for protecting these organelles and also cells against injuries having this indolamine a very high capacity. Moreover, melatonin has strong neuroprotective effects including properties to inhibit mitochondrial cytochrome c release and ensuing caspase activation [48,49,50,51]. We showed that melatonin administration in RIM rats supports antioxidant response in skeletal muscle and in blood serum, thus reducing FMS symptoms. In particular, we underlined that melatonin, restoring physiological levels of CoQ10 and the other proteins that we studied, is able to maintain mitochondrial homeostasis and to increase skeletal muscle resistance to injuries (Figure 4).

Therefore, mitochondria targeted antioxidants such as melatonin can be of scientific interest, and they should be taken in consideration for improving mitochondria health and/or mitochondrial related diseases [17,52]. In particular, the strong point of melatonin is due to the higher efficacy in mitochondria comparison to plenty kinds of antioxidants that have limited access to the same organelles. This point of view is reported also by Ramis et al. [53] suggesting that mitochondria-targeted antioxidants accumulate in several hundred-fold greater concentrations within mitochondria and protect these critical organelles from oxidative damage. Moreover, taking into consideration the production of melatonin within the mitochondria, it is possible to suggest that it has, for this reason, a very high antioxidant effect in these organelles and also in the cells.

## 4. Materials and Methods

### 4.1. Animal Treatment

Thirty-four Sprague Dawley male rats (4–5 weeks old) were housed in standard cages located in temperature-controlled animal facility with a 12h/12h light-dark cycle and free access to standard chow and tap water. The animals were randomly distributed in the following experimental groups:

- control rats kept untreated;
- control rats treated with melatonin for two months;
- control rats treated with the vehicle of melatonin;
- control rats treated with vehicle of reserpine;
- reserpine-induced myalgic rats (RIM);
- RIM rats plus tap water for two months (RIM + H_2_O);
- RIM rats treated with melatonin for two months (RIM + MEL).

Details on RIM model and melatonin preparation have been previously reported by Favero et al. [23]. In brief, reserpine, dissolved in 0.5% glacial acetic acid (vehicle), was injected subcutaneously once a day for three consecutive days at a final dose of 1 mg/kg body weight [9,54,55]. The treatment with melatonin (Melapure™ kindly provided by Flamma S.p.A., Chignolo d’Isola, BG, Italy), in combination or not with reserpine, was dissolved in ethanol (vehicle) and then administered orally for two months at the final dose of 5 mg/kg body weight/day. As we demonstrated previously, melatonin has dose- and time-dependently beneficial effects and the dose of 5 mg/kg body weight/day and two months of treatment presented the main observed protective effects in RIM [23].

The animals of all experimental groups were monitored for weight gain, food and water consumption and spontaneous motor activity carried out every day. Voluntary running activity was assessed in polycarbonate cages with free access to stainless steel activity wheels (Bioseb, In Vivo Research Instruments, Vitrolles, France). The wheel (diameter 23 cm; width 5 cm) could be turned in both direction and it was connected to an analyzer that automatically recorded the running activity. In particular, in the present study, we evaluated the distance traveled (expressed in meters) and the locomotor speed (expressed in meters/minute). No experimenters were present in the room during the recording period. Rats were habituated in an individual activity cage for three sessions over at least three days. A baseline measurement was recorded one day after the last habituation.

At the end of the treatments period, the animals were killed by decapitation and the gastrocnemius muscles were collected. The gastrocnemius muscles were adequately processed for morphometrical, ELISA and immunofluorescence analyses. In detail, for the morphometrical and immunofluorescence analyses, skeletal muscle samples were rapidly extracted and fixed in 4% buffered paraformaldehyde for 24 h, paraffin embedded and then sectioned using a microtome (7 µm of section thick) [23,56,57]. Whereas a small part of gastrocnemius samples of each experimental animal was adequately proceeded for ELISA assay, as then described in detail.

All the protocols were approved by the Animal Care and Use Committee of the University of Brescia (Brescia, Italy—DGSAF0006605—16/03/2015) and by the Italian Ministry of Health (558/2015-PR—22/06/2015) and comply the commonly accepted ‘3Rs’ indication (replacement, refinement, and reduction).

### 4.2. Morphometrical Analyses

Serial paraffin gastrocnemius sections were stained with hematoxylin-eosin and the sections were then observed with a light microscopy at final magnification of 400× (Olympus, Hamburg, Germany). Feret’s diameter of 50 myotubes for each animal gastrocnemius was determined using an image analyzer (Image Pro Premier 9.1, Media Cybernetics, Rockville, MD, USA). Two blinded investigators performed the morphometrical analysis and their evaluation was assumed to be correct if the values were not significantly different. If there was disagreement concerning the interpretation, the case was reconsidered to reach a unanimous agreement.

### 4.3. Immunofluorescence Assay

Serial paraffin gastrocnemius sections were deparaffined, rehydrated and then incubated in specific serum for one hour at room temperature. Then the sections were incubated one hour at room temperature and overnight at +4 °C with the following primary antibodies: mouse antibody against myogenin (diluted 1:200; Abcam, Cambridge, UK); rabbit antibody against PGC-1α (diluted 1:250; Abcam, Cambridge, UK); mouse antibody against Mfn2 (diluted 1:200; Abnova, Taipei City, Taiwan). After washing, the sections were labelled with 488 anti-mouse or 546 anti-rabbit Alexa Fluor conjugated secondary antibodies (diluted 1:200; Invitrogen, Paisley, UK). Finally, the sections were counterstained with 4′-6-diamidino-2-phenylindole (DAPI) [58,59,60], mounted and observed with a fluorescent microscopy (i50 Eclipse, Nikon, Düsseldorf, Germany) at final magnification of 400× [61,62]. Sections without primary antibody and in the presence of isotype-matched IgG served as negative immunofluorescent controls.

The immunopositivity for each primary antibody was quantified using an image analyzer (Image Pro Premier 9.1, MediaCybernetics Inc., Rockville, MD, USA) and expressed in arbitrary units (AU). Two blinded investigators performed the analysis and their evaluation was assumed corrected if the values were not significantly different. If there was disagreement concerning the interpretation, the case was reconsidered to reach a unanimous agreement [63,64].

### 4.4. Coenzyme Q10 ELISA Evaluation

Small pieces of gastrocnemius samples were homogenized, ultrasonicated and then centrifugated 15 min at 1500× *g*. The supernatants obtained were subjected to the analyses of the concentrations of CoQ10 through a specific ELISA assay kit (My Biosource, Inc., San Diego, CA, USA). The optical density values were determined using a microplate reader set at 450 nm (Sunrise, Tecan; Männedorf, Switzerland) and the CoQ10 values are expressed as ng/mL.

### 4.5. Statistical Analysis

Results were expressed as the mean ± standard error of the mean (SEM). Data for multiple variable comparisons were analyzed by one-way analysis of variance (ANOVA corrected Bonferroni test). *p* ≤ 0.05 was considered significant for all statistical analysis in this study.
